# Developing a core outcome set for people living with mild cognitive impairment: Protocol for the MCI Core Outcomes Study

**DOI:** 10.1002/alz.094949

**Published:** 2025-01-09

**Authors:** Victoria Grace Gabb, Natalie Woodward, Sam Harding, Angus McNair, Julie Clayton, Sophie Alderman, Winsome Barrett‐Muir, Alan Richardson, Sarah Rudd, Joseph Webb, Jemima Dooley, Elizabeth Coulthard, Nicholas Turner

**Affiliations:** ^1^ University of Bristol, Bristol United Kingdom; ^2^ North Bristol NHS Trust, Bristol United Kingdom; ^3^ NIHR Bristol Biomedical Research Centre, University of Bristol, Bristol United Kingdom; ^4^ University of Bath, Bath United Kingdom; ^5^ ReMemBr Lived Experience Expert Group, Bristol United Kingdom

## Abstract

**Background:**

Core outcome sets (COS) are a set of outcomes which stakeholders agree should be collected and reported as part of a minimum dataset for studies in a clinical population. COS developed collaboratively with patients and other stakeholders encourage researchers to report outcomes which are considered important by affected individuals. COS may reduce heterogeneity of outcome selection allowing for better synthesis across studies (e.g., meta‐analysis) and reduce selective reporting of positive outcomes to support more informed healthcare decisions. There is currently no approved COS for patients with mild cognitive impairment (MCI). Therefore, we are collaborating with patient and public contributors to develop a COS for people with MCI.

**Method:**

The study will involve three stages: 1) an umbrella review, which will provide outcomes from published studies by retrieving articles from systematic and scoping reviews of interventional studies involving people with MCI; 2) interviews with stakeholders with lived experience of MCI (people living with MCI and their partners/relatives) and relevant professional stakeholders (e.g., health and social care professionals or those from relevant charitable organisations or industry) will identify additional outcomes stakeholders consider important; 3) a “long list” of possible outcomes will be curated from Stage 1 and 2 and a two‐round Delphi survey process and consensus meeting(s) will identify the final COS to be recommended.

**Result:**

The study is ongoing. The review is at the data extraction stage. From the first 25 studies, the most common outcome measurement instruments reported so far are all related to cognitive functioning: the Mini‐Mental State Examination (9/25, 36%), Trail Making Test (6/25, 24%), and Verbal Fluency Test (6/25, 24%). Updated results will be available for the conference. Interviews are anticipated to start in mid‐2024 with the COS published in 2025.

**Conclusion:**

A COS for people with MCI will identify outcomes of importance for individuals affected by MCI and improve transparent outcome reporting in interventional research to better support patient care.

